# Evaluation of In Vitro Neuronal Protection by Postconditioning with Poloxamer 188 Following Simulated Traumatic Brain Injury

**DOI:** 10.3390/life11040316

**Published:** 2021-04-06

**Authors:** Luise J. Meyer, Matthias L. Riess

**Affiliations:** 1 Department of Anesthesiology, Vanderbilt University Medical Center, Nashville, TN 37232, USA; Department of Anesthesiology, University Medicine Greifswald, 17475 Greifswald, Germany; luise.j.meyer@gmail.com; 2 Anesthesiology, TVHS VA Medical Center, Nashville, TN 37212, USA; Department of Anesthesiology, Vanderbilt University Medical Center, Nashville, TN 37232, USA; Department of Pharmacology, Vanderbilt University, Nashville, TN 37232, USA

**Keywords:** cell membrane stabilizer, compression injury, hypoxia reoxygenation injury, ischemia reperfusion injury, neurons, P188, tri-block copolymer

## Abstract

Traumatic brain injury (TBI) leads to morbidity and mortality worldwide. Reperfusion after ischemia adds detrimental injury to cells. Ischemia/reperfusion (I/R) injures cells in a variety of ways including cell membrane disruption. Hence, methods to improve endogenous membrane resealing capacity are crucial. Poloxamer (P) 188, an amphiphilic triblock copolymer, was found to be effective against I/R and mechanical injury in various experimental settings. The aim of this study was to establish an in vitro mouse neuronal TBI model and, further, to investigate if *post*conditioning with P188 directly interacts with neurons after compression and simulated I/R injury, when administered at the start of reoxygenation. Cellular function was assessed by cell number/viability, mitochondrial viability, membrane damage by lactated dehydrogenase (LDH) release and FM1-43 incorporation as well as apoptosis-activation by Caspase 3. Five hours hypoxia ± compression with 2 h reoxygenation proved to be a suitable model for TBI. Compared to normoxic cells not exposed to compression, cell number and mitochondrial viability decreased, whereas membrane injury by LDH release/FM1-43 dye incorporation and Caspase 3 activity increased in cells exposed to hypoxic conditions with compression followed by reoxygenation. P188 did not protect neurons from simulated I/R and/or compression injury. Future research is indicated.

## 1. Introduction

Representing the most common cause of debilitating illness and mortality in young adults, traumatic brain injury (TBI) is a highly important concern in health care [1,2]. Each year, millions of people sustain TBI, leading to mental and physical debilitation, as well as death [1,2,3]. Current treatments for TBI are restricted to a few pharmacological and surgical therapies [2]. Therefore, in vitro and in vivo studies are crucial to determine potential targets for pharmacological strategies. While in vivo models better mimic the complexity of TBI in the clinical context, in vitro studies allow focusing on the pathophysiology and can be performed on individual cell types [4]. Among others, transection, compression, hydrostatic pressure, fluid shear stress, shear strain and stretch injury have been described as methods to simulate the components of TBI in vitro [4].

During TBI, the contusion to the head results in a primary lesion with an intracranial edema. Consequently, the intracranial pressure (ICP) rises due to the limited intracranial space [2]. Compression can be performed on cell lines to simulate the collision of the head and the elevated ICP. In accordance to the increased ICP, the cerebral perfusion decreases and causes ischemia [2]. To simulate the secondary ischemia in models of TBI, hypoxia works as a tool. Not only ischemia, but also the restoration of blood flow to the brain (e.g., reperfusion, reoxygenation) injures the tissue. Ischemia/reperfusion (I/R) injury describes the pathologies involved in the tissue damage precipitated by ischemia and subsequent reperfusion [5,6]. It presents a clinical conflict, since reperfusion is essential, but leads to additional harm. It is of utmost importance to develop strategies that prevent this incremental injury by reperfusion.

The amphiphilic copolymer Poloxamer (P) 188 has been shown to be protective in I/R injury models in the heart [7,8,9] and brain [8,10,11,12,13], including in vivo and in vitro TBI models [14,15,16,17,18,19]. More precisely, P188 was observed to attenuate neuronal cell death, membrane rupture, lactate dehydrogenase (LDH) leakage and activation of apoptosis in in vitro simulations of I/R [10,11]. Besides the protective effect of the copolymer in stroke models, P188 also preserved neuronal health in in vitro experiments of TBI [14,16,17,18,20,21,22,23]. P188 offered in vitro neuroprotection, amongst other, through cell viability increase, reduction in LDH release, apoptosis and necrosis, and improved morphological appearances after mechanically induced TBI with fluid shear stress [16,17,18,20,21,22]. Generally, P188 has been observed to seal membrane leakage, thus, preventing cell damage and death [8,23,24,25].

In the present study, the overall objective was to simulate TBI on primary isolated murine neurons by exposure to hypoxia (humidified 0.01% O_2_, 5% CO_2_, 94.99% N_2_, serum- and glucose-free media) and compression (1300 g/0.126 cm^2^, 1012 kPa), with subsequent reperfusion in normal culture conditions ± P188 (or polyethylene glycol [PEG]) treatment. Until now, only Glass et al. used mechanical injury by stretching and hypoxia [26]. Compared to their approach, we not only used compression instead of stretching but also applied it with hypoxia simultaneously throughout the complete injury time and not only in the beginning [26]. This was done to simulate the pathophysiology of TBI as closely as possible. Moreover, previous in vitro studies have mostly investigated P188 as a *pre*conditioning agent [10,15,27,28], which is not a suitable model to mimic the clinical setting; thus, current research tends to focus on administrating P188 as a *post*conditioning agent at the onset of reperfusion. Thereby, the present study aimed to provide new insights into TBI simulation in vitro and focused on a possible attenuation of I/R injury by P188 with conceivably direct reparative effects on neurons.

The overarching goal of the present study was to verify the hypothesis that the amphiphilic triblock copolymer P188 protects neurons against hypoxia/reoxygenation (H/R) and compression injury by stabilizing the injured cell membrane. Thus, we aimed at:

(1) Establishing a TBI model using compression and H/R to simulate I/R injury that leads to a moderate damage, still allowing P188 to attenuate cellular injury.
(2) Determining a protective effect of P188 when administered at the start of reoxygenation and using the completely hydrophilic polymer PEG as an osmotic control to elucidate if any P188 effect would be based on its amphiphilic character.

## 2. Materials and Methods

No human or animal research was performed; thus, no ethical approval was necessary.

### 2.1. Cell Culture

**Figure 1:**
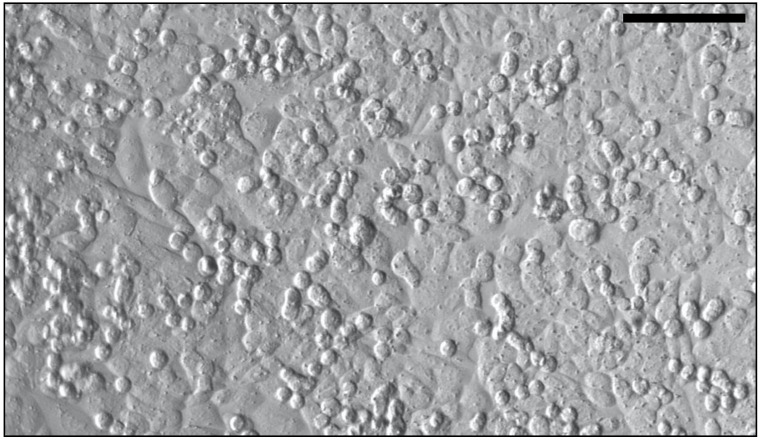
Representative image of isolated neurons from mouse brain cortex (20× magnification, Leica Inverted Laboratory Microscope). Scale bar represents 100 μm.

Isolated primary neuronal cell cultures from mouse brain cortex (Figure 1), as well as the culture media, passaging solutions, flasks and plates precoated with extra-cellular matrix for cell adherence were obtained from Celprogen (Torrance, CA, USA). Vials of neurons (lot # 18060501) were purchased and shipped frozen to be used for all experiments of this study. According to manufacturer’s specifications, cells were cultured in neuronal growth media containing Fetal Bovine Serum (FBS) and antibiotics and incubated in a standard culture environment of humidified 21% O_2_, 74% N_2_, 5% CO_2_ at 37 °C. Carried out under these conditions, Celprogen assured that the biochemical and molecular properties of the isolated neurons are analogue to those of in vivo neurons. To support neuronal growth and proliferation, neurons were treated with 20% FBS by adding 50 mL FBS (Gibco by Thermo Fisher Scientific, Waltham, MA, USA) to the complete growth media (500 mL) already containing 10% FBS. The neurons expressed a number of neuronal markers, including neurofilaments, microtubule associated protein 2, Beta III tubulin, neuron specific enolase 2 and Tau protein. The neurons contained a majority of attached adherent cells and a few floating cells in suspension. Due to the fast growth with a doubling time of 24 h, the neurons were sub-cultured for cell expansion when grown to confluency. The cells were used for up to 22 passages.

For experiments, neurons between passages 6–22 were plated at seeding densities of 70,000 and 80,000 cells/cm^2^ in clear as well as black-walled clear-bottom, 96-well plates and placed in standard culture environment. Experiments were generally performed between 48 and 72 h after plating, when neurons reached confluency. This was done to mimic the normal in vivo physiological state of the cells. Two replicate plates were plated for each endpoint assay within one experiment, because one plate underwent normoxic conditions ± compression, whereas the other hypoxic conditions ± compression.

### 2.2. In Vitro Simulation of Traumatic Brain Injury

**Figure A1:**
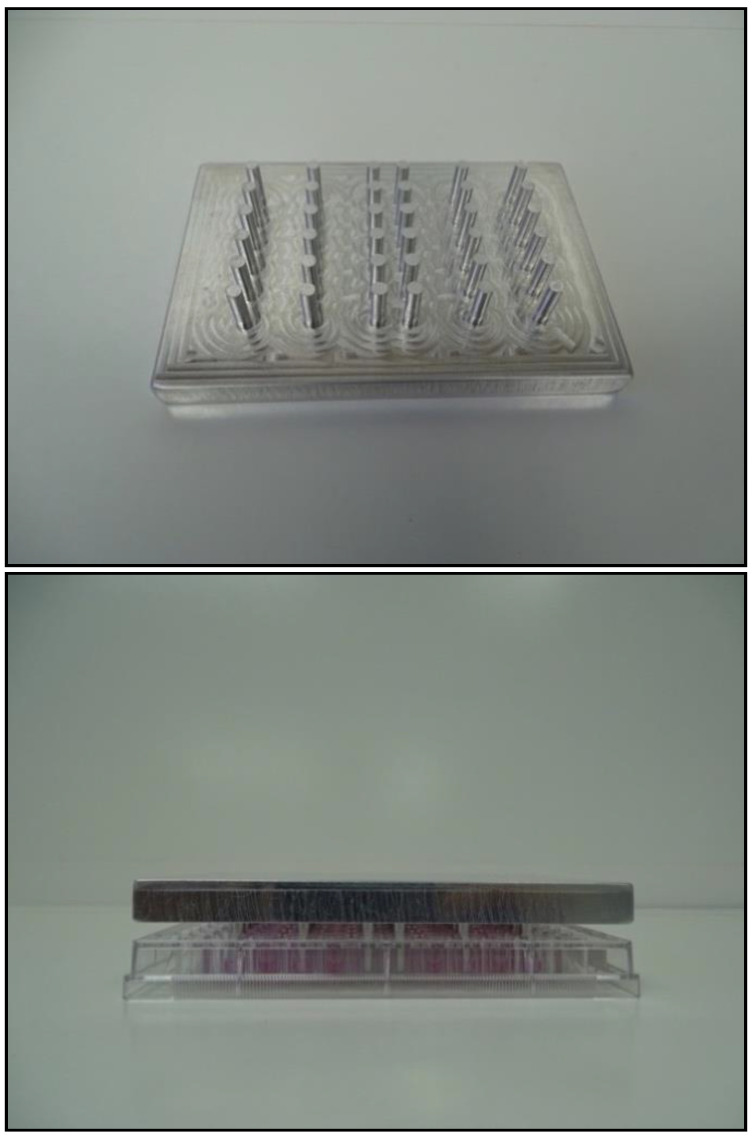
Image of aluminum compression lids: rods (15 mm length × 4 mm diameter) compress the neurons in the plate. We designed aluminum lids that matched the 96-well plates with rods which fit in the wells to compress the neurons (custom-made at the Vanderbilt University Medical Center Machine Shop). An additional weight of 1000 g was put on the 300 g empty weight aluminum lids to injure the cells appropriately. Accordingly, 1300 g/0.126 cm^2^ with a pressure of 1012 kPa affected the neurons within one well.

**Figure A2:**
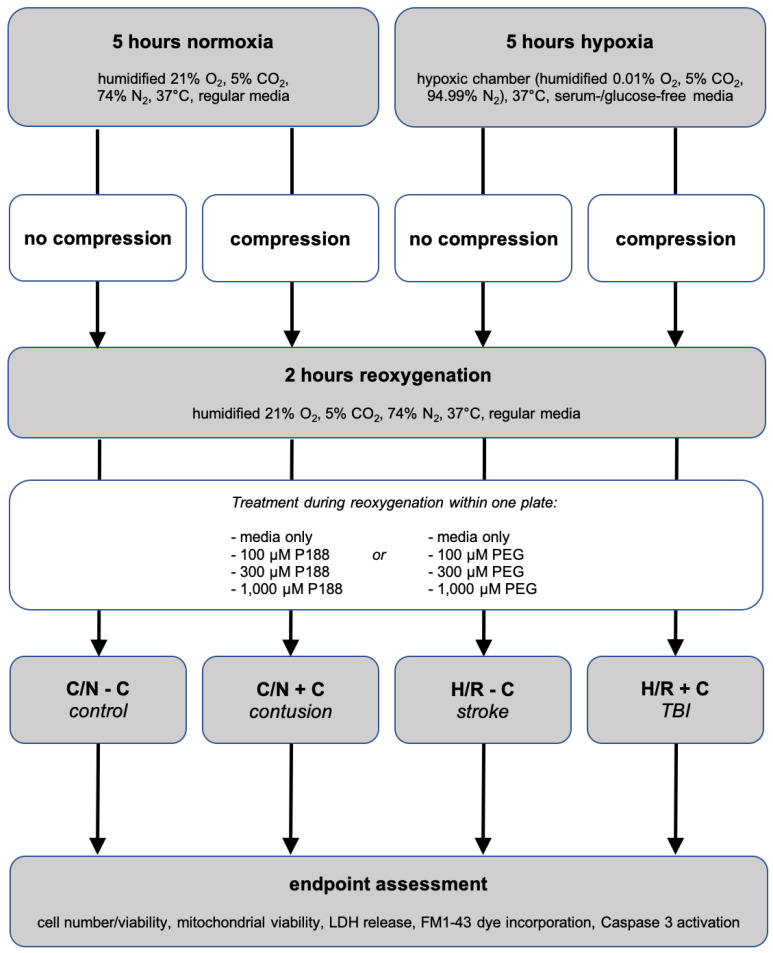
Flow chart of experimental set-up: Four different conditions were investigated: control/normoxia without compression (C/N-C) and with compression (C/N+C), as well as hypoxia/reoxygenation without compression (H/R-C) and with compression (H/R+C). C/N-C is considered as control, whereas C/N+C mimics contusion, H/R-C stroke, and H/R+C traumatic brain injury (TBI) in vitro. Within each condition, four different concentrations of Poloxamer (P)188 or polyethylene glycol (PEG) were tested: no treatment (media only), 100 µM P188/PEG, 300 µM P188/PEG and 1 mM P188/PEG. LDH: lactate dehydrogenase.

As we have previously presented at international meetings [29,30,31], we developed an in vitro model to simulate TBI with exposure of cells to compression and H/R to mimic I/R injury in vitro. Therefore, we designed special compression lids (Figure A1), put them on top of the experimental plates containing neurons and placed them in a hypoxia chamber (Stemcell Technologies; Vancouver, BC, Canada). More precisely, as shown in Figure A2, plates with confluent neurons were randomly divided to normoxic conditions in regular media and standard environment or hypoxic conditions in a hypoxia chamber and cultivated in serum- and glucose-free media. Conditions in the hypoxia chamber were humidified 0.01% O_2_, 5% CO_2_, 94.99% N_2_. In addition, compression was performed under both normoxic and hypoxic conditions to achieve 4 groups: Control/normoxia (C/N) without compression (C/N-C) (1) and with compression (C/N+C) (2) as well as H/R without compression (H/R-C) (3) and with compression (H/R+C) (4). H/R+C is considered an in vitro simulation of TBI, whereas H/R-C mimics stroke. C/N+C can be interpreted as a contusion-like scenario in vitro, whereas C/N-C is considered the control group.

Compression and hypoxia were performed simultaneously for 5 h (for dose finding, see Section 2.3 and Section 3.1). This was followed by 2 h of reoxygenation: the experimental plates containing neurons were taken out from the hypoxia chamber, the compression lids were removed, the media was replaced with regular media containing serum and glucose, and the plates were returned to standard culture conditions of humidified 21% O_2_, 74% N_2_, 5% CO_2_ at 37 °C. To control for any influences of media exchange, the normal media of C/N cells was replaced at the same time as the hypoxic media was changed to the normoxic media for reoxygenation.

At the start of reoxygenation, cells were treated with different concentrations of P188, as well as PEG for the entire reoxygenation period (cf. Figure A2, Section 2.4).

### 2.3. Model Development via Optimization of H/R ± Compression Duration

To optimize the H/R ± compression protocol with mouse neurons, a series of experiments to injure the neurons were performed. We evaluated 2.5, 5 and 10 h of hypoxia ± compression, followed by 2 h of reperfusion as successfully accomplished before [32]. In this context, we were able to demonstrate that 5 h hypoxia ± compression with subsequent 2 h of reoxygenation works as a suitable in vitro mouse neuron TBI model for testing novel treatments [31].

### 2.4. Treatment with P188 and PEG

The effect of P188 on mouse neurons was tested at concentrations of 100 μM, 300 µM, and 1 mM. At concentrations ranging from 100 µM to 1 mM, P188 and PEG are ≥96% soluble, do not affect the pH value of the media, and exist in a dynamic solution of single molecules and grouped micelles [33].

Furthermore, the selected concentrations have been established in previous studies investigating P188. For example, P188 in concentrations ranging from 10 µM to 1 mM, administered during reoxygenation, protected mouse cardiomyocytes from I/R injury assessed by cell number/viability, LDH release, FM1-43 incorporation, Caspase 3 activity and intracellular Ca^2+^ [32]. The effect of P188 has also been tested on murine myoblast cell membrane protection after exposure to hypotonic stress and isotonic recovery followed by evaluation of membrane integrity via LDH release. Herein, 14 µM P188 was needed to reduce LDH release to half [34]. Moreover, 150 µM P188 fully ameliorated sarcomere length stretch injury in dystrophin deficient cardiac myocytes [35]. In addition, in a mouse neuronal hippocampal stroke model in vitro, P188 in concentrations ranging from 1 µM to 1 mM reduced cell death, LDH leakage and preserved plasma membrane integrity provoked by I/R [10].

Analogous to P188, the same concentrations for PEG were examined for appropriate osmotic control. Both P188 and PEG—dissolved in the media at the selected concentrations—were administered as *post*conditioning agents at the beginning of 2 h of reperfusion.

### 2.5. Endpoint Assessments

Neuronal endpoint assessment was performed by several well-established indices of cellular function (cell number/viability, mitochondrial viability, membrane damage by LDH release/FM1-43 dye cell incorporation and apoptosis initiation by Caspase 3 activity) at the end of each experiment, thus, after 2 h of reperfusion.

Cell number and viability were assessed using the PrestoBlue^®^ Cell Reagent (Invitrogen by Thermo Fisher Scientific, Waltham, MA, USA). The cells were washed twice with 100 µL phosphate-buffered saline (PBS)/well (Sigma-Aldrich, St. Louis, MO, USA). Next, 90 μL of media were added, then 10 μL of the PrestoBlue^®^ Cell Reagent. The cells were incubated for 30 min at 37 °C in a humidified incubator. Using a plate reader (Synergy H1 Microplate Reader, BioTek Instruments, Inc., Winooski, VT, USA), the fluorescence of each well was read at an excitation (Ex) of 560 nm and emission (Em) of 590 nm (bottom read). Data are expressed as average number of vital cells per well.

Mitochondrial viability was assessed using the colorimetric CellTiter 96^®^ AQueous One Solution Cell Proliferation Assay (Promega, Fitchburg, WI, USA). The cells were washed twice with 100 µL PBS/well. Then, 20 μL of the CellTiter 96^®^ AQueous One Solution Reagent was added in each well containing fresh 100 μL of media and cells and incubated for 1–1.5 h in a humidified, 5% CO_2_ atmosphere. The absorbance was recorded at 490 nm with a top-reading plate reader. Data were expressed as average absorbance units per well, with “well” equaling the average number of cells determined in the plate used to assess cell number/viability within the same experiment.

Cellular injury was assessed by the colorimetric measurement of the intracellular enzyme LDH using the Pierce LDH Cytotoxicity Assay Kit (Thermo Fisher Scientific, Waltham, MA, USA). Based on impaired cell membrane integrity, LDH leakage by injured cells from the cytoplasm into the surrounding culture media is measurable in the cell culture media. To estimate the LDH release, two measurements had to be done: analysis of the LDH in the supernatant media/well and LDH in the lysed cells/well. 50 μL sample/well (out of 100 µL culture media/well) were transmitted to the equivalent well of a new 96-well clear plate and mixed with 50 μL of prepared reaction mixture. After 30 min incubation at room temperature protected from light, the reactions were terminated by addition of 50 μL stop solution. The absorbance of formazan, which displays the released LDH, was then measured at 490 nm using a plate reader (top read) to determine the LDH in the supernatant media/well. To assess the LDH in the lysed cells/well, the original experiment plate containing cells and the remained 50 µL media were lysed. To do this, 10 μL of Lysis Buffer (10×) were added to the primary clear 96-well plate containing cells with 50 μL of media left and incubated at 37 °C, 5% CO_2_ for 45 min. The signaling absorbance readings were too high for our plate reader sensitivity, and the samples were, therefore, diluted with a dilution factor of five. A fraction of 10 μL/well was mixed with 40 μL of PBS and 50 μL of reaction mixture and transferred in a new 96-well plate. After incubation at room temperature for 30 min protected from light, 50 μL stop solution were added, and the absorbance was measured at 490 nm. The assay results had then to be multiplied with the dilution factor five. The LDH leakage was calculated as follows: LDH release per total = LDH in culture media ÷ LDH in cell lysate. Data are expressed as average absorbance of LDH in culture media per absorbance of LDH in cell lysate.

Cell membrane injury was assessed using the membrane impermeant styryl dye FM1-43 (Molecular Probes, Inc., Eugene, OR, USA). This compound is impassable to intact cell membranes. When the cell membrane is damaged, the fluorescent FM1-43 dye is incorporated into the lipid bilayer of the cell membrane [32,35,36]. It has been used to assess membrane damage and reparation in various cell types [32,35]. Following the media change at the start of the reoxygenation period, 5 µM of FM1-43 was added to the media. As required for reoxygenation, the cells were then incubated for 2 h. Prior to endpoint readings, non-internalized FM1-43 that remained extracellular was washed out using 100 µL PBS and replaced by 100 µL of fresh media with serum. The fluorescence was read at Ex = 488 nm and Em = 568 nm using a bottom-reading plate reader. Data was expressed as average relative fluorescent units (RFU) per well, with “well” referring to the corresponding well used to assess cell number/viability within the same experiment.

The Caspase 3/CPP32 Fluorometric Assay Kit (BioVision, Inc., Milpitas, CA, USA) was used to examine the Caspase 3 activity as an indicator of apoptosis. The synthetic substrate DEVD-AFC (AFC: 7-amino-4-trifluoromethyl coumarin) emits fluorescence upon cleavage by Caspase 3. Cells were resuspended in 50 μL of chilled cell lysis buffer. Cells were incubated on ice for 10 min, and afterwards 50 μL of 2× Reaction Buffer (containing 10 mM Dithiothreitol [DTT]) were added. DEVD-AFC with an amount of 5 μL of 1 mM (50 μM final concentration) were additionally added and plates were incubated for 1–2 h at 37 °C. The fluorescence was read at Ex = 400 nm and Em = 505 nm using a bottom reading plate reader. Data was expressed as average relative RFU per well, with “well” referring to the corresponding well used to assess cell number/viability within the same experiment.

### 2.6. Cellular Morphology Assessment

Cellular morphology after different injury mechanisms was imaged using an inverted laboratory microscope (Leica DM IL LED), the CCD Microscope Camera (DFC365 FX) and the software platform for life science LAS X Life Science, version 2.0 (Leica Microsystems, Inc., Buffalo Grove, IL, USA).

### 2.7. Statistics

Data are presented as percentages, normalized to the condition C/N-C. In every experiment, for each endpoint assay, the absolute values were normalized to the values of C/N-C (without P188/PEG treatment). Therefore, C/N-C was set to 100% within one experiment per endpoint.

All resulting data were tested for normality with the Shapiro-Wilk test and for equal variance via Brown-Forsythe. If both tests were passed (*p* > 0.05), data analyses were performed through parametric testing. If one or both of them failed (*p* < 0.05), nonparametric testing was performed.

For parametric testing, one-way-Analysis of Variance (ANOVA) was conducted to compare the means of different groups. Not normally distributed values were expressed as median (25th; 75th quartile). Kruskal-Wallis One Way ANOVA on Ranks testing was performed on non-parametric data.

Post hoc testing was applied to significant results. Differences were considered statistically significant with *p* < 0.05 (two-tailed). After Kruskal-Wallis, Dunn’s Method for All Pairwise Multiple Comparison procedures was used as post hoc testing. If data were normally distributed (Shapiro-Wilk), but the Equal Variance Test (Brown-Forsythe) failed, data were expressed as median (25th; 75th quartile) and Kruskal-Wallis with Dunn’s Method was performed as well.

If the normality test (Shapiro-Wilk), as well as the equal variance test (Brown-Forsythe) were passed, Student-Newman-Keuls Method for All Pairwise Multiple Comparison Procedures was applied.

Graphically, brackets indicate significance (*p* < 0.05) between different conditions without treatment, and * expresses significance vs. the same condition without treatment. All data were analyzed with SigmaStat 4.0 (San Jose, CA, USA).

## 3. Results

### 3.1. Model Development

Previously, we compared durations of hypoxia ± compression of 2.5, 5 and 10 h with subsequent 2 h reoxygenation to develop our neuronal TBI in vitro model [31]. Samples were assayed for cell number/viability, membrane damage by LDH release per total and mitochondrial viability. Herein, we were able to demonstrate, that hypoxic injury remained time-dependent, whereas compression injury was not. While 2.5 h hypoxia injured the neurons insufficiently and 10 h excessively, 5 h hypoxia still led to a significant damage, but moderate enough to allow an additional increase through compression [31], and further potential attenuation by protective strategies. Therefore, 5 h hypoxia ± compression with 2 h reoxygenation, appeared to be a suitable in vitro mouse neuron TBI model for testing novel treatments and has been used upon this study.

### 3.2. Changes in Neuronal Morphology after Injury

**Figure A3:**
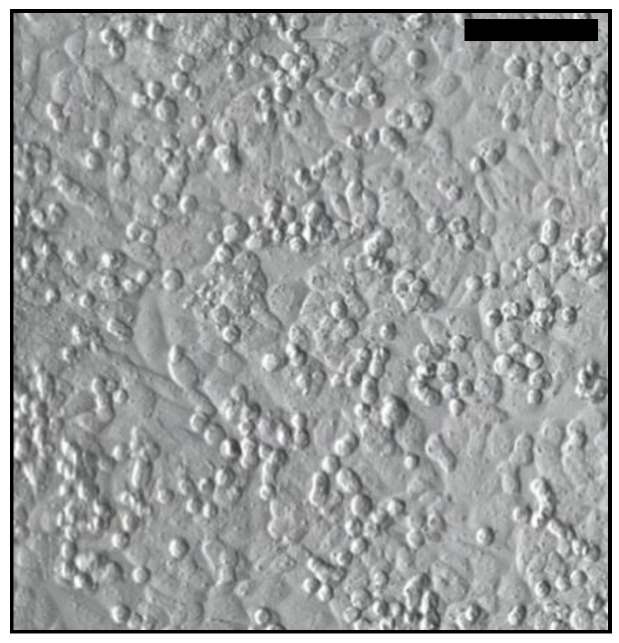
Subjective description of neuronal morphology before injury (20× magnification, Leica Inverted Laboratory Microscope): confluent cells impose as cuboidal when attached to ground, as well as rounded up when floating above. Scale bar represents 100 μm.

**Figure A4:**
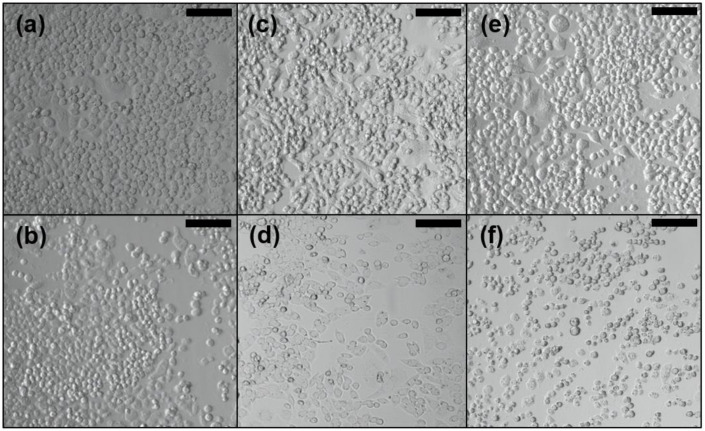
Subjective description of neuronal morphology of neurons after exposure to different injury mechanisms (20× magnification, Leica Inverted Laboratory Microscope): (**a**) 5 h compression; (**b**) 5 h compression/2 h reoxygenation (C/N+C); (**c**) 5 h hypoxia; (**d**) 5 h hypoxia/2 h reoxygenation without compression (H/R-C); (**e**) 5 h hypoxia and compression; (**f**) 5 h hypoxia and compression/2 h reoxygenation (H/R+C); compression (**a**,**b**): icosahedron similar shape; hypoxia (**c**,**d**): fusiform/spindle-shaped cells; compression and hypoxia (**e**): massive rounding of neurons; after reoxygenation (**b**,**d**,**f**) cells lost confluency, rounded up; H/R+C (**f**) few shrunk and round cells, almost no contact. Scale bars represent 100 μm.

The effect of injury was also subjectively evaluated by assessing cell morphology. Uninjured neurons, when grown to confluency, exhibited two different morphologic shapes (Figure A3): Some neurons were—to a certain extent—attached to the surface and thereby of cuboidal morphology, whereas others were more rounded, floating on top of other cells. After neurons were exposed to compression for 5 h (Figure A4a), their shape resembled an icosahedron, with less contact to each other. Hypoxia, in contrast, led to a more fusiform/spindle-shaped morphology of the neurons attached to the surface with many neurons bubbling and rounding up, providing less contact to each other (Figure A4c). When hypoxia and compression were combined for 5 h, many rounded-up and some fusiform-shaped cells could be observed (Figure A4e). Remarkably, after additional 2 h of reoxygenation, cells looked even more injured under all 3 conditions (Figure A4b,d,f), with a huge loss of confluency and cell contact observable. After hypoxia and reoxygenation without compression (H/R-C) (Figure A4d), cells exhibited a fusiform character; after compression and reoxygenation (C/N+C) (Figure A4b) cells rounded up even more compared to compression with no reoxygenation afterwards. After hypoxia and compression with subsequent reoxygenation (H/R+C) (Figure A4f), cell body shrinkage and rounding-up was detectable with the greatest deficit in confluency.

### 3.3. Effects of P188 on Simulated Traumatic Brain Injury in Neurons

#### 3.3.1. Cell Number and Viability

**Figure 2:**
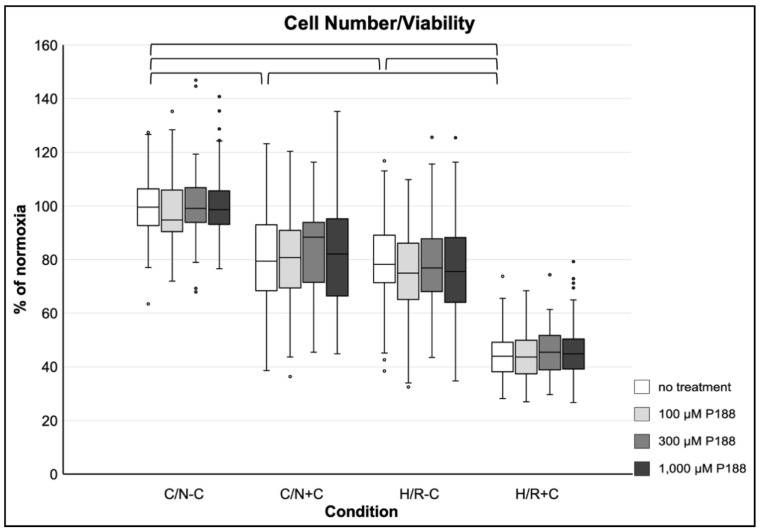
Cell number/viability after applied injury and P188 treatment. C/N: control/normoxia; H/R: hypoxia/reoxygenation; ±C: with/without compression; C/N-C with no treatment (normoxia) equals 100%; brackets indicate *p* < 0.05 between conditions without treatment; *n* = 12 experiments per group.

There was a significant decrease in cell number/viability of approximately 20% after 5 h H/R-C, as well as after compression alone (C/N+C), compared to cells under C/N-C conditions. After H/R combined with compression (H/R+C), there was a nearly 60% decrease of cell number/viability. No significant effect of P188 was observed throughout all normoxic and hypoxic conditions with or without compression on cell number/viability (Figure 2).

#### 3.3.2. Mitochondrial Viability

**Figure 3:**
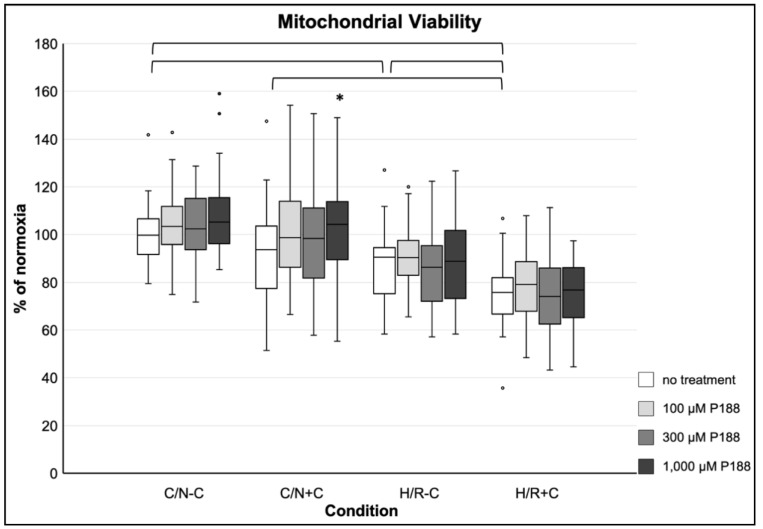
Mitochondrial viability after applied injury and P188 treatment. C/N: control/normoxia; H/R: hypoxia/reoxygenation; ±C: with/without compression; C/N-C with no treatment (normoxia) equals 100%; brackets indicate *p* < 0.05 between conditions without treatment; * vs. no treatment, same condition; *n* = 8 experiments per group.

Compression alone (C/N+C) did not result in a significant decrease of mitochondrial viability compared to C/N-C. H/R-C slightly decreased mitochondrial viability compared to C/N-C, whereas the combination of H/R and compression (H/R+C) led to a 25% decline of mitochondrial viability. Generally, no positive or negative effects of P188 were observed on mitochondrial viability, except for a slight, but statistically still significant increase after 1000 µM P188 in C/N+C conditions compared to no treatment (Figure 3).

#### 3.3.3. Membrane Damage as Assessed by LDH Release per Total

**Figure 4:**
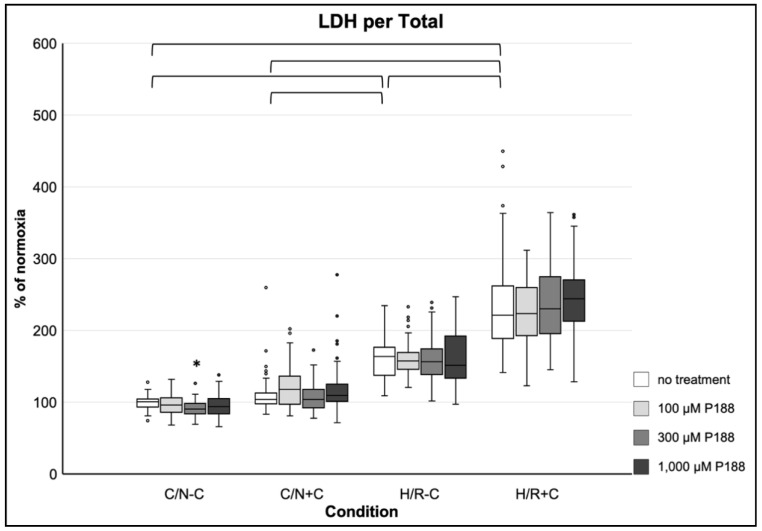
LDH release per total after applied injury and P188 treatment. C/N: control/normoxia; H/R: hypoxia/reoxygenation; ±C: with/without compression; C/N-C with no treatment (normoxia) equals 100%; brackets indicate *p* < 0.05 between conditions without treatment; * vs. no treatment, same condition; *n* = 8 experiments per group.

Compression alone (C/N+C) did not result in a significant increase of membrane damage, whereas hypoxia (H/R-C) did. The combination of both (H/R+C) resulted in an even greater damage. A more than two-fold increase of LDH release per total after H/R+C was observed compared to C/N-C. No beneficial effect of P188 on LDH release per total was observed. However, a mild, but significant decrease of LDH release per total in 300 µM P188 compared to no treatment was detectable in the C/N-C condition (Figure 4).

#### 3.3.4. Membrane Damage as Assessed by FM1-43 Dye Incorporation

**Figure 5:**
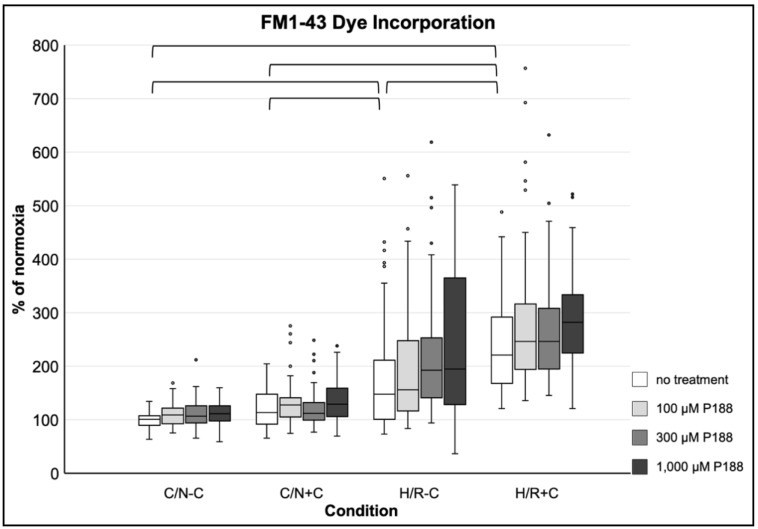
FM1-43 incorporation after applied injury and P188 treatment. C/N: control/normoxia; H/R: hypoxia/reoxygenation; ±C: with/without compression; C/N-C with no treatment (normoxia) equals 100%; brackets indicate *p* < 0.05 between conditions without treatment; *n* = 8 experiments per group.

Compression (C/N+C) alone did not result in a significant FM1-43 dye incorporation, whereas hypoxia (H/R-C) induced membrane damage as assessed by FM1-43 dye incorporation. The combination of both hypoxia and compression (H/R+C) resulted in even greater membrane damage. A more than two-fold increase of FM1-43 dye incorporation compared to C/N-C was observed after H/R+C. Throughout all normoxic and hypoxic conditions with or without compression, no significant effect of P188 was observed on FM1-43 dye incorporation (Figure 5).

#### 3.3.5. Induction of Apoptosis

**Figure 6:**
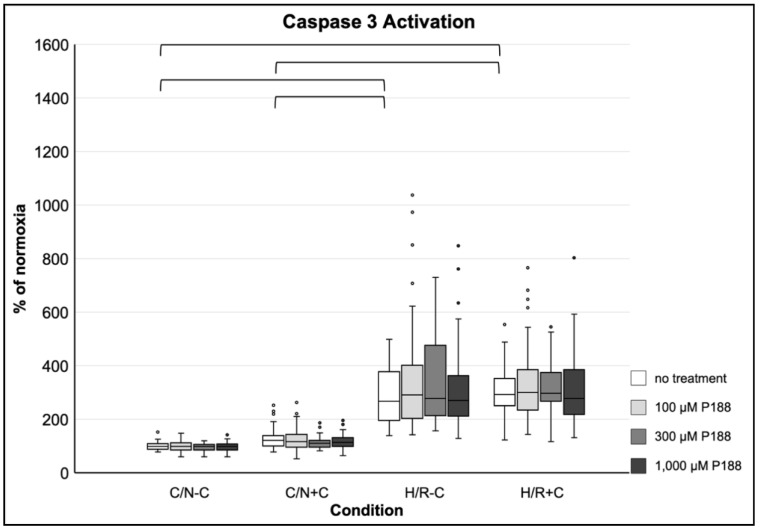
Caspase 3 activity after applied injury and P188 treatment. C/N: control/normoxia; H/R: hypoxia/reoxygenation; ±C: with/without compression; C/N-C with no treatment (normoxia) equals 100%; brackets indicate *p* < 0.05 between conditions without treatment; *n* = 8 experiments per group.

H/R-C as well as H/R+C increased Caspase 3 activity almost three-fold, whereas no statistical significance between C/N-C and C/N+C was observed. No significant effect of P188 was found on Caspase 3 activity throughout all normoxic and hypoxic conditions with or without compression (Figure 6).

### 3.4. Effects of PEG on Simulated Traumatic Brain Injury in Neurons

#### 3.4.1. Cell Number and Viability

**Figure A5:**
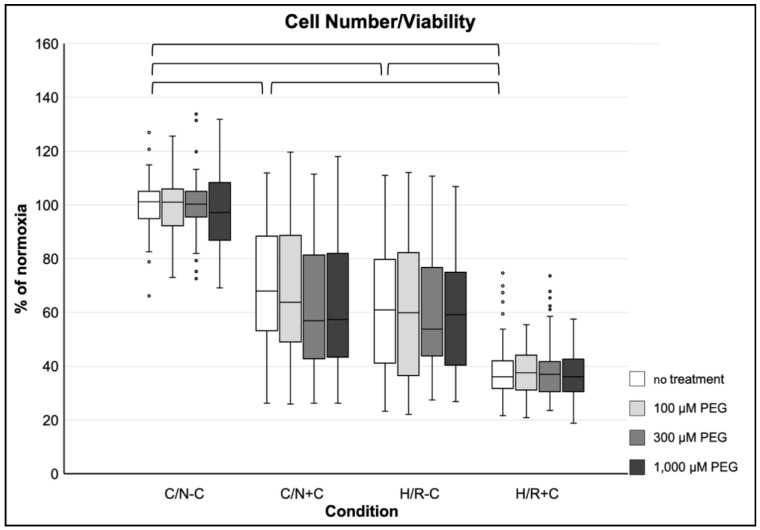
Cell number/viability after applied injury and PEG treatment. C/N: control/normoxia; H/R: hypoxia/reoxygenation; ±C: with/without compression; C/N-C with no treatment (normoxia) equals 100%; brackets indicate *p* < 0.05 between conditions without treatment; *n* = 12 experiments per group.

There was a significant decrease of approximately 30% in cell number/viability after 5 h compression (C/N+C), as well as a 40% decrease after 5 h hypoxia (H/R-C), compared to cells under C/N-C conditions. After H/R+C there was a nearly 60% decrease of cell number/viability detectable. PEG did not attenuate cell number/viability, nor showed it any negative effect on cell number/viability (Figure A5).

#### 3.4.2. Mitochondrial Viability

**Figure A6:**
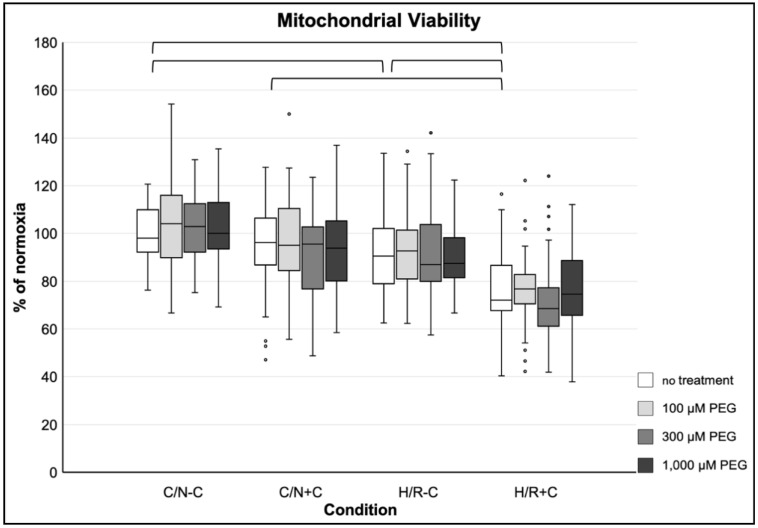
Mitochondrial viability after applied injury and PEG treatment. C/N: control/normoxia; H/R: hypoxia/reoxygenation; ±C: with/without compression; C/N-C with no treatment (normoxia) equals 100%; brackets indicate *p* < 0.05 between conditions without treatment; *n* = 8 experiments per group.

Compression alone (C/N+C) did not result in a significant decrease of mitochondrial viability compared to C/N-C. H/R-C decreased mitochondrial viability by nearly 10% compared to C/N-C, whereas H/R+C led to an approximate 25% decline of mitochondrial viability. Generally, neither protective nor harming effects of PEG were observed on mitochondrial viability (Figure A6).

#### 3.4.3. Membrane Damage as Assessed by LDH per Total

**Figure A7:**
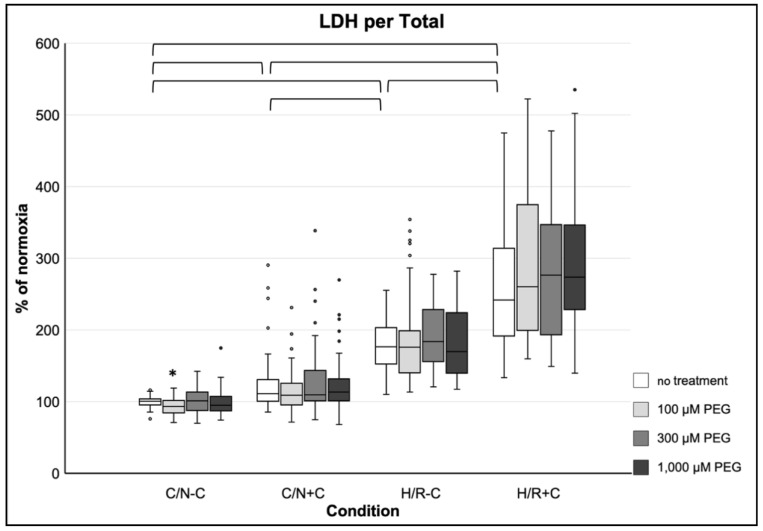
LDH release per total after applied injury and PEG treatment. C/N: control/normoxia; H/R: hypoxia/reoxygenation; ±C: with/without compression; C/N-C with no treatment (normoxia) equals 100%; brackets indicate *p* < 0.05 between conditions without treatment; * vs. no treatment, same condition; *n* = 8 experiments per group.

Compression (C/N+C) showed an approximate increase of LDH release per total to 110%, whereas hypoxia (H/R-C) led to an increase of LDH release per total to nearly 180% compared to control. The combination of both (H/R+C) potentiated the damage. H/R+C increased LDH release per total to approximately 240% compared to C/N-C conditions. Nearly no influence on the damage was found by PEG, except for a slight, but significant decline of LDH release per total after treatment with 100 µM PEG compared to no treatment in the C/N-C condition (Figure A7).

#### 3.4.4. Membrane Damage as Assessed by FM1-43 Dye Incorporation

**Figure A8:**
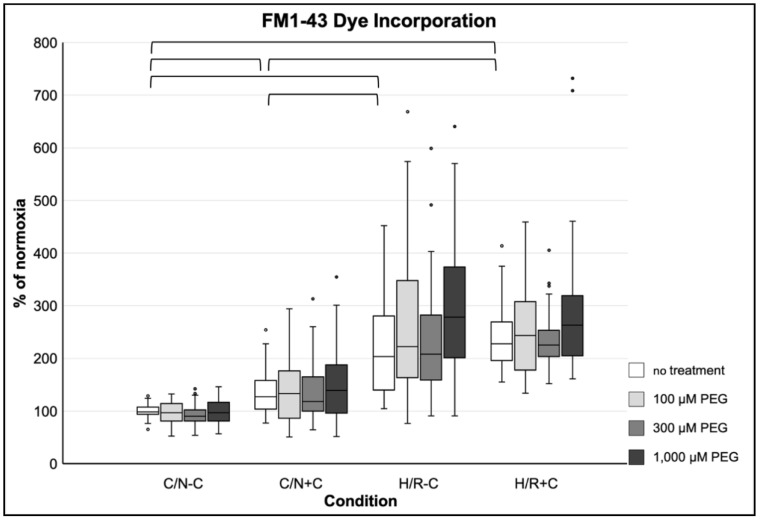
FM1-43 incorporation after applied injury and PEG treatment. C/N: control/normoxia; H/R: hypoxia/reoxygenation; ±C: with/without compression; C/N-C with no treatment (normoxia) equals 100%; brackets indicate *p* < 0.05 between conditions without treatment; *n* = 8 experiments per group.

Compression alone (C/N+C) did result in a slight, but significant FM1-43 dye incorporation increase, whereas hypoxia (H/R-C) induced a two-fold higher FM1-43 dye incorporation. The combination of both (H/R+C) resulted in greater membrane damage up to nearly 230% compared to C/N-C conditions, assessed by the incorporation of the fluorescent styryl dye. No significant difference between the damage induced by hypoxia alone (H/R-C) and hypoxia paired with compression (H/R+C) could be observed. Neither a positive nor a negative effect of PEG was observed throughout all normoxic and hypoxic conditions with or without compression (Figure A8).

#### 3.4.5. Induction of Apoptosis

**Figure A9:**
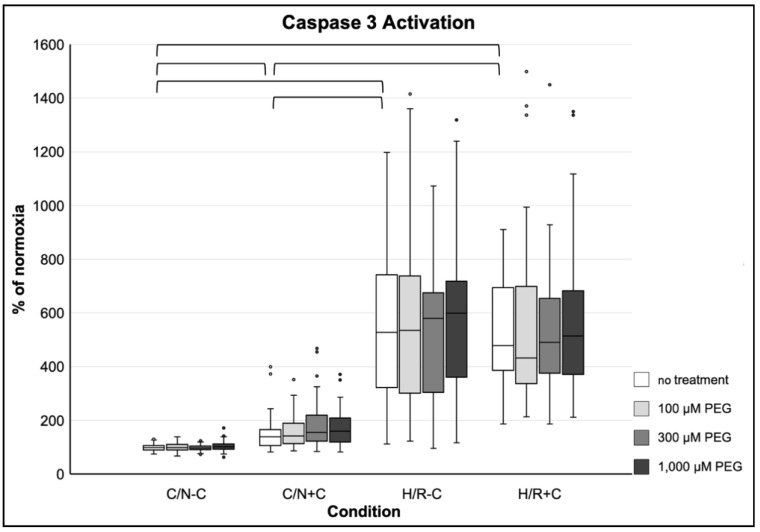
Caspase 3 activity after applied injury and PEG treatment. C/N: control/normoxia; H/R: hypoxia/reoxygenation; ±C: with/without compression; C/N-C with no treatment (normoxia) equals 100%; brackets indicate *p* <0.05 between conditions without treatment; *n* = 8 experiments per group.

Compression alone (C/N+C) increased Caspase 3 activity to nearly 140%. Hypoxia (H/R-C), instead, increased Caspase 3 activity by more than five-fold, whereas H/R+C by almost five-fold. No significant difference in apoptosis activation between H/R-C and H/R+C was seen. Neither a positive nor a negative effect of PEG could be observed (Figure A9).

## 4. Discussion

Oxygen deprivation in the brain occurs during ischemic stroke or after suffering TBI. The restoration of blood flow and oxygen supply to ischemic tissue (reperfusion, reoxygenation), while necessary, aggravates the ischemic injury itself. The complex pathophysiological mechanisms underlying these circumstances are summarized as I/R injury [5,6]. Reperfusion remains critically important as it demonstrates the earliest possibility of beginning the therapy of TBI and stroke.

Focusing on the patho-mechanism of TBI, the effect of H/R (simulated I/R) and compression injury as components of TBI was investigated on primary mouse brain neurons in vitro. This injury protocol caused significant damage to the neurons, including a decrease in cell number/viability and mitochondrial viability, activation of apoptosis by Caspase 3, as well as an increase in membrane damage assessed by LDH release per total and FM1-43 dye incorporation, when compared to cells under C/N conditions. More precisely, hypoxia alone (H/R-C) as well as its combination with compression (H/R+C) injured the cells, although their combination (H/R+C) exerted a more severe damage when assessed by cell number/viability, mitochondrial viability and LDH per total. Thus, the neuronal damage examined by these three endpoints seemed to be achieved by hypoxia or compression alone and lead to an increased damage when combining the two. In contrast, Caspase 3 activity did not increase through the addition of compression, indicating that activation of apoptosis—while achieved through hypoxia—may not be triggered by compression injury. It might be concluded, that compression exerts its damage too severely to activate apoptosis, but instead leads to neuronal cell death via necrosis. Hypoxia, in turn, might allow induction of controlled cell death. No precise statement could be made about FM1-43 dye incorporation as additional dye incorporation after H/R+C compared to H/R-C was observed in the P188 data (Figure 5), but not in the PEG data (Figure A8). Under C/N conditions, compression exerted its damage more inconsistently so that not all assays and data sets (P188 and PEG data) showed concordant statistically significant differences between C/N+C and C/N-C. While this may be attributable to the limited number of experiments, compression might just exert less damage than hypoxia. Moreover, cellular morphology altered, especially upon reoxygenation, following hypoxia ± compression, illustrating the demand for investigating strategies to attenuate reperfusion injury.

Hence, we administered the amphiphilic copolymer P188, a membrane sealant known to stabilize membranes in I/R injury [8,9,10], at the start of reoxygenation in different concentrations. The nontoxic, nonionic triblock copolymer P188 consists of two hydrophilic portions (polyethylene oxide [PEO] chains) that surround a hydrophobic center (polypropylene oxide [PPO]). This hydrophobic part is proposed to plug membrane damages, thus, sealing membrane leakage and ensuring intact membranes and cell viability [8,24,25,35,37]. Further, we applied the completely hydrophilic polymer PEG as it is considered the ideal control molecule for P188. The PEG we used has a similar molecular weight [38] and, therefore, similar osmotic properties as P188 [32,39]. Thus, it is suited to test the hypothesis that P188’s membrane sealing effects are due to its unique amphiphilic character and not due to its osmotic properties.

Contrary to our expectations, P188 administered at the start of reoxygenation did not attenuate neuronal cell injury in our model. This was objectified by the five aforementioned end point assays and 3 logarithmic concentrations of P188 and PEG. The scarce statistical significances after P188 treatment compared to no treatment only appeared under normoxic conditions, were just marginal (cf. Figure 3 and Figure 4, Figure A7), and are, therefore, interpreted as negligible. As no relevant differences between the groups of P188 treatment and no treatment, as well as PEG treatment have been observed, it can be concluded that P188 was not able to restore neuronal vitality after induced injury in vitro.

### 4.1. Effect of P188 on Neuroprotection

A considerable amount of literature has been published on neuroprotective properties of P188 in stroke and TBI injury models [8,10,11,13,14,15,16,17,18,19,20,21,22,23,27,28,40,41,42,43,44,45,46,47,48,49,50]. There is evidence that P188 attenuates cerebral I/R and mechanical injury amongst others through improvement of cell membrane integrity, provision of a functional impermeable blood-brain barrier (BBB), reduction in neuronal cell death, as well as suppression of apoptosis [8,10,15].

Despite these promising results, no beneficial effect of P188 was apparent in our present *post*conditioning model. The compound did neither increase cell viability or mitochondrial viability, nor did it diminish membrane degradation or LDH release per total, nor did it reduce the rate of programmed cell death/apoptosis.

In order to comprehend why P188 might not have protected neurons in our present model, it is important to examine studies where P188 positively affected neurons, to illustrate the differences between the models. To begin with, in the present study, only an in vitro model of H/R paired with compression injury in isolated neurons was used. In contrast, in vivo studies have revealed neuroprotective effects of P188 [10,11,13,15,19,27,28,40,41,43,44,45,48,49,50], thus, identifying the potential importance of the physiological condition and environment of the brain with all its cell components as well as tissue perfusion.

However, P188 also protected neurons against excitotoxicity and oxidative injury [23], mechanically induced stress [14,16,17,18,20,21,22] and oxygen and glucose deprivation [10,11] in vitro. Furthermore, a recent review compared the therapeutic efficiency of compounds from in vivo and in vitro studies in which it was demonstrated that in vitro studies are suitable to predict in vivo models and are accordingly effective to test novel treatments [4].

Further demonstrating this point, Gu et al. [10] deprived HT22 mouse hippocampal neuronal cells from oxygen and glucose, and reoxygenated them thereafter to simulate I/R injury in an in vitro stroke model. P188 administration in concentrations ranging from 1 µM to 1 mM reduced cell death, LDH leakage and preserved plasma membrane integrity [10]. However, there are several differences to our present model that are important to note. Firstly, Gu et al. investigated hippocampal neurons [10], whereas we focused on primary neurons derived from the cortex. However, the same concentrations of P188 administered in the present study were used by Gu et al., and the simulation of I/R injury was executed similarly—as in our present model, H/R was achieved by glucose deprivation and exposure to hypoxia in a hypoxia chamber and subsequent reoxygenation under normal culture conditions. Unlike 5 h of hypoxia followed by 2 h of reoxygenation as in the present study, Gu et al. exposed the hippocampal cells to hypoxia for 18 h, and reoxygenated them thereafter for 24 h. The biggest difference to the present model, however, is that P188 administration was performed 1 h before oxygen and glucose deprivation until the end of reoxygenation. Hence, hippocampal cells were exposed to P188 for 43 h (in contrast to 2 h of reoxygenation in the present experimental set-up). Thus, as P188 was applied as a preventive measure, it does not surprise that P188 attenuated cell injury in Gu et al.’s experiments. It might also be possible that P188 needs to be exposed to cells for a longer duration to exhibit its membrane sealing properties.

Similarly, Bao et al. treated mice injured with TBI with P188 30 min before cortical impact and revealed that this pretreatment reduced lesion size and BBB permeability. Moreover, brain edema, neurological deficits, neuronal cell death and apoptosis were attenuated [15]. This strengthens the argument that in our present study, it might not have been possible to observe a membrane sealing effect by P188 because P188 was only administered upon reoxygenation. Nevertheless, our present approach depicts a more clinically applicable situation, since cerebral ischemia is not a foreseeable event, unless it occurs under very specific circumstances such as vascular or neurosurgery.

On the other hand, numerous studies, in which P188 has been only administered upon reperfusion/reoxygenation were still able to demonstrate neuroprotection [11,13,14,16,17,18,19,20,21,22,23,40,41,43,44,45,50]. For example, our study group, as well as Luo et al. were able to show that after 90 min or 60 min, respectively, of middle cerebral artery occlusion in rats, treatment with P188 upon beginning of reperfusion was able to reduce infarct size and rescue locomotor function post-ischemia [11,13]. Additionally, Zhang et al. administered Vepoloxamer (purified P188) 2 h post-TBI in rats, which still provided improved functional outcome as well as neuroprotection and anti-inflammation [19]. Both of these studies, however, were conducted in vivo.

In conclusion, it is technically possible to achieve a neuroprotective effect of P188 in models of TBI and stroke. However, in the *post*conditioning in vitro model discussed here, no beneficial properties of P188 on isolated primary neurons could be observed. This might be due to the use of an in vitro model and/or treatment timing (*pre*- vs. *post*conditioning). Additionally, it is simply explainable by the fact that P188 might not exert its beneficial effect directly on neurons, because the model per se did induce appropriate injury.

In this context, it remains unclear if brain cells other than neurons, such as glial cells—specifically astrocytes, oligodendrocytes, ependymal cells and microglia/macrophages—or brain vascular endothelial cells, do mediate the neuroprotection obtained by P188. Indeed, P188 has protected both astrocytes and brain endothelial cells in in vitro settings of blast-induced TBI [42,46,47]. Furthermore, we were able to reveal endothelial neuroprotection by P188 in an experimental setting close to the present model with compression and I/R injury in vitro [29]. P188 was additionally observed to reduce ischemia-induced BBB permeability in vivo [10,15,50]. The BBB consists of neurons, but also of endothelial cells, the foot processes of astrocytes and pericytes, as well as cellular elements like tight junctions and the basal lamina [15,50,51]. This further supports the hypothesis that neurons might not represent a target in the neuroprotection mediated by P188, but rather other brain cells. P188 did not only diminish BBB permeability, but also reduced brain edema development in a rat model of TBI. In this regard, the downregulation of Aquaporin 4 by P188 was assessed, which is located on astrocytes and endothelial cells [15], thus, indicating the potential role of both these cell types in the attenuation of brain edema by P188. In investigations of Langendorff-isolated rat hearts with P188 treatment, increasing concentrations of P188 resulted in an elevated nitric oxide (NO) production. Furthermore, this inducible effect was completely abolished by NO synthase inhibition [52]. NO synthase inhibition also abolished the functional improvement and infarct size reduction by P188 in ischemic Langendorff-isolated rat hearts [53]. The protective effect of P188 may be therefore mediated by NO production [52,53]. The vasodilatory signaling molecule NO, that is important for vascular homeostasis, is produced by endothelial cells [54], further strengthening the hypothesis that endothelial cells play a part in P188’s cell protection.

Recently it has been demonstrated that P188 ameliorates dopaminergic neurodegeneration in the substantia nigra and alleviates hippocampal synapse loss, as well as behavioral impairment—which might be caused by microglial phagocytic activity—in a neurotoxicity-induced mouse model of Parkinson’s disease. Microglia activation might be a therapeutic target of P188 not only in Parkinson’s disease but also in other neurological disorders [55]. Consistently, it has been also reported that P188 has a neuroprotective and anti-inflammatory effect by reduction of microglia/macrophage activation and astrogliosis in the brain after TBI in rats [19].

Taken together, there is evidence that P188 might not exert neuroprotection directly through neurons, but rather through microglia, astrocytes and/or endothelial cells in the brain.

### 4.2. Study Limitations

The present study naturally does have limitations, which need to be considered to accurately review and discuss our results:

- The use of merely an in vitro TBI model of H/R ± compression injury represents both a strength and a limitation. In vitro experiments are indispensable in basic research as they provide a tool in the commencement of investigating novel therapeutic strategies and help identify pharmacological targets [56,57]. However, the use of an in vitro protocol generally implies that the applied mechanism to generate cellular damage might not represent the actual pathophysiological state of injury in a living body [56].
- The investigation of a primary culture—developed of immature tissue—might not appropriately simulate adult TBI. Primary neuronal cultures were harvested from newborn C57B/6 mouse tissue [58]. Thus, it could be argued that experiments on primary neurons might not be optimal for mimicking adult TBI but rather for investigations of the infantile brain. Indeed, neurons derived from embryonic tissue do not exhibit glutamate-mediated excitotoxicity because they do not express glutamatergic receptors. Excitotoxicity, however, plays an important role in adult neuronal injury. Moreover, for harvesting primary cultures mechanical and enzymatic dissociation processes are necessary, which can already lead to cellular disruption and injury [57].
- Brain tissue not only consists of neurons but also of other cells like glial and endothelial cells [56,59], making the use of primary neurons out of their environment only partially representative.
- The compression with heavy aluminum lids and H/R injury used in our model might not be adequate for attenuation by P188. Different techniques to simulate TBI in vitro have been considered in other studies [4], some of which might be better-suited to study membrane resealing properties and, consequently possible positive effects of P188 on neuronal regeneration after TBI. TBI models with fluid shear stress have been observed to exert neuronal damage that can be abolished by P188 [16,17,18,20,21,22]. One might suggest that the injury induced by shear stress might be more adequate for investigating P188’s neuroprotective properties, possibly due to a shorter duration of damage and perhaps different cellular targets that might experience injury. Thus, the model used in the present investigation, while effective, might not have injured the neurons in a way that can be alleviated by P188.
- In the present model, cells in the center of a well suffered more mechanical injury than cells at the edge of a well due to the placement of the rods of the compression lids. Hence, it is possible that cells—especially in the center of a well—were excessively and irreversibly injured by compression, thus, making it possible that the injury was beyond what can be attenuated by potential sealing strategies. However, cells that did not undergo compression could also not be rescued by P188. Yet, it is additionally possible that the applied H/R injury was too excessive for being alleviated by P188.
- Neurons were only treated with P188 as a *post*conditioning agent after 5 h of injury. It is possible that an earlier beginning of P188 treatment could have allowed a better neuronal outcome. This hypothesis is supported by previous investigations: Serbest et al. observed a neuroprotective effect of P188 after injuring neuronal cells for a short duration (200 ms), and, consequently, an earlier P188 administration, already after 15 min; furthermore, cells were treated with P188 for 24 h [17,18]. Another study by Gu et al. achieved neuroprotection through a 43-h exposure to P188 [10]. Exposing the cells to P188 for no longer than 2 h, as in the present study, might not have been enough time for the neurons to regenerate. However, previous investigations of H/R and compression injury in different cells have revealed a protective effect of P188 after only 2 h of reoxygenation [29,32,60,61].
- No proven positive control/neuroprotectant was examined against this type of injury to show the general ability to protect neurons in this model. However, while P188 failed to attenuate neuronal cell injury, we have no evidence to suggest that our model would preclude other neuroprotective agents from being effective.
- Cell morphology changes have been described subjectively. Systematic quantitative assessment has been outside the scope of the present work. Yet, the observations made are concordant with the results of our objective endpoint assays (cell number/viability, mitochondrial viability, LDH, FM1-43 dye incorporation, Caspase 3 activation) and would not have changed our conclusions.
- Based on previous work of our study group, we have chosen 2 h for reoxygenation [32]. We did not further explore different reoxygenation times as this was outside the scope of our current work.

Briefly, one might think that only mildly injured cells may recover with the help of P188. However, the fact that P188 was not able to rescue even less injured neurons that only underwent H/R without compression, raises the possibility that P188 does not directly interfere with neurons at all.

Nevertheless, it is also conceivable that the injury used here cannot be diminished by the membrane sealant P188. As it is not possible to ascertain if the exerted H/R and compression damage in this study extends to more than just membrane leakage, it is possible that P188 did not protect neurons because it is proposed to only exert neuroprotection through sealing membranes. It is, therefore, possible that neurons injured with only membrane damage—caused in a different and more controlled manner—could be saved by P188 and that, especially in in vivo studies and in clinical trials, P188 might exert more robust neuroprotective properties.

### 4.3. Future Directions

If neurons are directly affected by P188’s membrane resealing properties remains to be elucidated. Future research should focus on identifying which cells are involved in the protective effect of P188. Prospective investigations should explore if brain cells other than neurons, such as glial cells—specifically astrocytes—or brain endothelial cells, do mediate the neuroprotection. As mentioned above, P188 has indeed protected both astrocytes and brain endothelial cells in in vitro settings of TBI [29,42,46,47] and was able to attenuate ischemia-induced BBB impermeabilities, specifically [10,15,50]. The impact of P188 on the BBB should therefore be pursued in a more detailed fashion.

The proposed form of brain preservation through P188 could be investigated via different cell culture systems and tissue preparations as alternatives to primary neuron culturing [57]. This could help to simulate a more physiological state of the brain through the interplay between different cell components. While, in this regard, co-culturing seems to be a simple approach, organotypic culturing—where thin brain slices are cultivated in vitro without cell dissociation [56,57]—may work as an even more physiologic model. In the same manner, three-dimensional (3D) cell culturing systems are of great interest. Here, cells are grown in 3D matrix gels to closely mimic physiological cell differentiation and organization, which is not possible in regular two-dimensional culturing [62]. Furthermore, for studying TBI simulation more approximately to in vivo, cerebral organoids can be used. Organoids are 3D in vitro culturing systems, that are grown from stem cells and can closely depict the architecture and functionality of in vivo tissue [63]. All these culturing systems will be important for the development of appropriate TBI simulations to, finally, discover a more defined pathophysiological mechanism of P188.

Finally, in vitro settings could extend to guide in vivo neuronal membrane protection through P188. Therefore, in vivo TBI studies with weight drop and subsequent P188 administration would be recommended to better simulate the pathophysiological mechanisms of TBI and to describe if P188 might work as a potential therapy towards I/R and compression injury.

P188 was observed to only weakly adsorb into the lipid bilayer, hypothetically provoked by the two PEO chains. This suggests that removal of one PEO chain alleviates and strengthens the PPO interaction with the lipid bilayer without losing the amphiphilic character [64]. In this subject, diblock copolymers (PEO-PPO structure) have been recently examined in regard to resealing properties on stressed myoblast membranes. The interaction of diblocks with the lipid bilayer has been described as an “anchor and chain” mechanism. Accordingly, diblocks that have a hydrophobic end group flanked to their PPO block are capable to anchor deeply into the cell membrane, thus, potentially sealing membranes with more strength than triblocks [8,34,64,65]. In this context, diblocks can be synthesized individually with different sizes and chemistry of end groups to ultimately refine membrane resealing. However, the interaction of diblock copolymers with injured neurons was outside the scope of the current work; yet, it represents a subject of increasing scientific inquiry.

## Data Availability

Data are available from the corresponding author upon specific request.
